# Intimate Partner Violence, Relationship Power Inequity and the Role of Sexual and Social Risk Factors in the Production of Violence among Young Women Who Have Multiple Sexual Partners in a Peri-Urban Setting in South Africa

**DOI:** 10.1371/journal.pone.0139430

**Published:** 2015-11-23

**Authors:** Yanga Z. Zembe, Loraine Townsend, Anna Thorson, Margrethe Silberschmidt, Anna Mia Ekstrom

**Affiliations:** 1 Health Systems Research Unit, Medical Research Council of South Africa, Cape Town, South Africa; 2 Department of Public Health Sciences /Global health, Karolinska Institutet, and Department of Infectious Diseases, Karolinska University Hospital, Stockholm, Sweden; 3 Department of Public Health, University of Copenhagen, Copenhagen, Denmark; University of Toronto Dalla Lana School of Public Health, CANADA

## Abstract

**Introduction:**

This paper aims to assess the extent and correlates of intimate partner violence (IPV), explore relationship power inequity and the role of sexual and social risk factors in the production of violence among young women aged 16–24 reporting more than one partner in the past three months in a peri-urban setting in the Western Cape, South Africa. Recent estimates suggest that every six hours a woman is killed by an intimate partner in South Africa, making IPV a leading public health problem in the country. While there is mounting evidence that levels of IPV are high in peri-urban settings in South Africa, not much is known about how it manifests among women who engage in concomitantly high HIV risk behaviours such as multiple sexual partnering, transactional sex and age mixing. We know even less about how such women negotiate power and control if exposed to violence in such sexual networks.

**Methods:**

Two hundred and fifty nine women with multiple sexual partners, residing in a predominantly Black peri-urban community in the Western Cape, South Africa, were recruited into a bio-behavioural survey using Respondent Driven Sampling (RDS). After the survey, focus group discussions and individual interviews were conducted among young women and men to understand the underlying factors informing their risk behaviours and experiences of violence.

**Findings:**

86% of the young women experienced IPV in the past 12 months. Sexual IPV was significantly correlated with sex with a man who was 5 years or older than the index female partner (OR 1.7, 95% CI 1.0–3.2) and transactional sex with most recent casual partner (OR 2.1, 95% CI 1.1–3.8). Predictably, women experienced high levels of relationship power inequity. However, they also identified areas in their controlling relationships where they shared decision making power.

**Discussion:**

Levels of IPV among young women with multiple sexual partners were much higher than what is reported among women in the general population and shown to be associated with sexual risk taking. Interventions targeting IPV need to address sexual risk taking as it heightens vulnerability to violence.

## Introduction

Every 6 hours a woman is killed by an intimate partner in South Africa, resulting in the world’s highest reported rate of intimate femicide [1]. Clearly, the violence that women in South Africa experience is not only gendered, but intimate and waged in the private sphere of women’s homes where gender norms that promote gender based violence are shaped and sustained. This type of violence is commonly referred to as intimate partner violence (IPV) [2], and refers to all acts of physical, sexual, psychological and emotional violence committed by a current or former intimate partner or spouse [3].

In the high HIV prevalence context of South Africa, where young women have a disproportionately higher prevalence of HIV than men in their age cohort (5.7% versus 0.7% among young women and men aged 15–19; and 5.1% versus 17.4% among young women and men aged 20–24) [4], IPV has been established as an independent risk factor for HIV [5]. The association between HIV and IPV is not unexpected since the two epidemics share a common risk environment characterized by poverty, limited economic opportunities, high-risk sexual norms, and gender and economic inequalities in South Africa [6]. These factors combine to undermine women’s capacity to prioritize and choose protective sexual behaviour on the one hand, while enabling the performance of masculine power that sustains high levels of violence on the other hand [7].

Other factors thought to be important for a complete understanding of the relationship between IPV and HIV include sexual risk behaviours such as multiple sexual partnering, transactional sex [8, 9, 10], and age mixing (having a partner who is 5 or more years older than the index female or male partner) [11]. Much of the evidence on the intersections between IPV and sexual risk behaviour among heterosexual populations in South Africa is informed by studies conducted among men and women in the general population [12, 13] rather than among those who are the most-at-risk of HIV infection, such as heterosexual women who have multiple sexual partners. The few studies that have been conducted among populations who report having multiple sexual partners suggest that they may have higher levels of IPV than sexually active men and women in the general population. For instance, in a recent study that we conducted among heterosexual men who have multiple sexual partners in a peri-urban community in the Western Cape, we found that they perpetrated IPV at much higher rates than have been reported by studies among heterosexual men in the general population [14]. Prior to our study, no studies of this kind had been conducted among women who have multiple sexual partners. Knowledge about the particular ways in which IPV intersects with other risk behaviours is needed to inform the development of tailored and effective interventions that address the manner in which sexual risk behaviours such as transactional sex and age mixing increase the risk of IPV among women who have multiple sexual partners in South Africa.

The association between IPV sexual risk taking is likely mediated by relationship power inequity, which refers to the degree to which decision-making power is unequally and unfairly distributed between sexual partners [15]. This type of power inequity is upheld by patriarchal social belief systems that support power differentials between men and women [5, 9]. It is further reinforced by age and economic asymmetries, which emphasize women’s economic limitations and their dependency on male sexual partners for material survival [5, 9, 16]. Yet, relationship power inequity is rarely measured in studies investigating IPV among women in South Africa [16]. The few studies that have measured it suggest that high relationship power inequity is significantly associated with more frequent experiences of IPV [5], low condom use [16] and HIV infection [8].

This paper aims to assess the extent and correlates of IPV, explore relationship power inequity and the role of sexual and social risk factors in the production of violence among young women aged 16–24 who have multiple sexual partners in a peri-urban setting in the Western Cape, South Africa. Women aged 15–24 years have disproportionately higher rates of HIV than their male counterparts, which suggests that they are infected by males in older age cohorts, which in turn suggests age mixing and the possibilities of transactional sex [4]. They are also more likely to experience IPV than older women [17]. It is for these reasons that young women aged 16–24 were selected as the study population for this research.

## Methods

### Study setting

The study was conducted in the Cape Winelands region, 60kms north of Cape Town, in the Western Cape Province in South Africa. The Western Cape Province has the highest rates of crime against women in the country [18] and among the highest rates of community violence in the world [19]. Rates of community and interpersonal violence are highest in the areas historically designated for occupation by Black and Coloured populations, i.e. townships [19]. The study community, a poor, Black, township is one such area. It has a population of 25,600 people, the majority of whom are Black (99%) and largely unemployed (38%) [20]. The prevalence of HIV in this community is not precisely known, but a recent bio-behavioural survey found a prevalence of 5% among women aged 16–24 who reported having multiple sexual partners [21]. The community has its own local epidemic of violence, with recent crime statistics showing an almost 50% increase in violent crime in the community between 2008/2009 and 2011/2012 [22]. Importantly, the community is a by-product of the Group Areas Act No. 41, of 1950, which made it compulsory for people to reside in areas declared for the exclusive use of their particular racial group only. Today this community is poor and marginalized from mainstream economic development. It is characterised by numerous, small government-issued homes, backyard shacks, and an ever increasing number of alcohol serving venues where gender based violence commonly occurs.

### Study design

Quantitative and qualitative methods were used to collect data between 2007 and 2009 from sexually active young women.

#### Quantitative methods

The quantitative data that form the basis of this paper were drawn from a larger bio-behavioral survey (BBS) among women who have multiple sexual partners. The BBS measured HIV prevalence and associated risk factors including IPV. In the quantitative study we used respondent driven sampling (RDS) to recruit young Black women who reported having multiple sexual partners in the past three months. RDS is effective for recruiting populations who engage in stigmatized behaviors and are thus hidden or hard-to-reach, and where a sampling frame is impossible to construct [23, 24]. The details of the RDS recruitment and data collection process are reported in detail elsewhere [21]. In the quantitative survey, eligible participants were female; aged 16–24 years; residing/ working/ socializing in the study community; self–reporting more than one male sexual partner in the past three months; and reporting friendship or acquaintance with one or more women who have multiple sexual partners. Our analytical sample comprised 259 young women.

Consenting young women who fulfilled the eligibility criteria completed a 103-item survey questionnaire that assessed experiences and the frequency of IPV, women’s attitudes regarding gender relations, relationship power inequity, demographic information and sexual risk behaviours.

IPV was measured using the WHO violence against women instrument [8]. Questions enquired about physical and sexual partner violence in the past 12 months. A typical question asked “In the past 12 months did your main partner slap you or throw something at you which could hurt you because you made him angry?”

Statements to assess women’s attitudes regarding gender relations were taken from a validated Likert scale used by Jewkes et al [5] in a cluster randomized trial implemented among a similar group of young women in the Eastern Cape, South Africa. The questions in the scale were asked using colloquial language. For an example one of the items asked if participants agreed or disagreed that “It is ok to hit a woman”.

To measure relationship power equity we used a validated sexual relationship power scale (SRPS) originally consisting of 23 items [5, 16] but reduced to 15 for the purposes of our study. The SPRS items enquired about decision-making dominance and relationship control [5, 16]. Typical items included statements such as “when my partner wants me to sleep over he expects me to agree”; “I would not be able to terminate my relationship even if I wanted to”; “when my main partner has given me money, he expects me to do everything that he wants me to do”, to which participants agreed or disagreed.

Demographic and sexual risk behaviour variables are defined and reported in greater detail elsewhere [21]. Questionnaires were self-completed in isiXhosa and English.

To analyze the survey data, estimates of population proportions and 95% confidence intervals (CIs) for all demographic variables, IPV outcomes and sexual risk behaviors were calculated using the Respondent-Driven Sampling Analysis Tool 5.6 (RDSAT) (www.respondentdrivensampling.org). RDSAT 5.6 enables analyses of equilibrium, and generates sample weights to take into account differential recruitment (homophily) and variations in participants’ network sizes [23].

We fit multiple logistic regression models to assess the correlates of IPV in the past 12 months, controlling for hypothesized confounders (age, socio economic status and education or school status), and including explanatory variables (transactional sex, age mixing, condom use, number of partners, concurrency, sexual debut and partner fidelity) and indicators of relationship power equity, women’s attitudes regarding gender relations and weights generated by RDSAT 5.6 on the main outcome variable—IPV. Explanatory variables with a p-value above 0.25 were removed by backward elimination, so that final models consisted of demographic characteristics and explanatory values where p ≤ 0.25.

#### Qualitative Methods

A year after the quantitative survey was completed, we returned to the study community to conduct a qualitative enquiry among young women to help us better understand the nature and underlying drivers of sexual risk behaviours and IPV. We chose to recruit women attending alcohol-serving venues known as shebeens due to the acknowledged association between visits to alcohol serving venues and several HIV risk factors including sexual risk behaviours [25, 26, 27], and IPV [28, 29]. We chose to recruit women of a similar age to those who completed the BBS, i.e. women age 16–24 years. Thus we used criterion-based, purposive sampling to recruit 36 sexually active young women aged 16–24, who were sober, residing in the study community, and at the selected alcohol serving venue at the time of data collection.

Consenting young women were invited to participate in one of four focus group discussions involving 8–10 participants each or 9 semi-structured individual interviews.

Following the same process described above, we also recruited 6 men aged 23–32 to participate in a focus group discussion about local men’s perceptions of sexual relationships, power, control and how they related to their female sexual partners.

We used open ended focus group discussion and interview guides that inquired about sexual behavior, violence, power and control in intimate relationships in the study community. All of the focus group discussions and interviews were conducted in isiXhosa, the language spoken by over 90% of residents in the study community. The focus group discussions and interviews were audiotaped and transcribed. The first author, a young, Black, Xhosa speaking, PhD student, and a locally trained, young, Black, Xhosa speaking female research assistant recruited all the female participants and co-facilitated the focus group discussions. However, only the first author conducted the individual interviews. Two Black, Xhosa speaking, male research assistants co-facilitated the men’s focus group discussion.

Data were analysed using Graneheim et al´s content analysis methods [30]. This process entailed identifying, coding and categorising the content of the transcripts into themes [31]. The analysis comprised two levels; first we conducted surface level identification and coding of the visible components of the text in the transcripts, this is referred to as manifest content analysis. At the second level we applied an interpretative analysis approach to understand the latent meaning of the text.

### Ethics statement

All of the participants gave informed, written consent prior to data collection. Due to the sensitivity of our research questions, parental consent for participants who were younger than 18 was not individually sought. Instead the lead author conducted parent information sessions in all the schools in the study community during which parents were asked if they would object if their daughters who were younger than 18 years of age participated in the study. No parents objected to the possible inclusion of their underage children in the study. The Research Ethics Committee of the Faculty of Health Sciences, University of Cape Town, South Africa granted ethics approval to conduct this inquiry.

## Results

### Demographic characteristics, IPV and sexual risk behaviours

**Fig 1:**
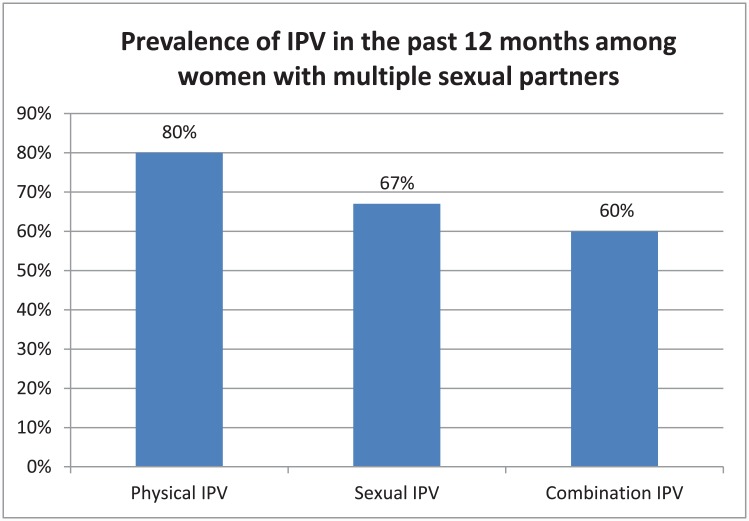
x axis: Types of intimate partner violence; y axis: Prevalence of each type of intimate partner violence.

Almost three quarters of the young women were aged 16–19 and school going (74%, CI 72–83). More than half of the participants (56%, CI 45–59) were poor and 21% (CI 17–27) were abjectly poor. Most of the young women (86%, CI 81–90) reported being beaten, slapped or forced to have sex by intimate partners in the past 12 months; some experienced both physical and sexual IPV (Fig 1); and 25% (CI 20–30) experienced IPV more than once in the past 12 months (Table 1).

**Table 1:** Demographics & Intimate partner violence sample and estimated population proportions with 95% confidence intervals.

Variable	N	Sample Proportions%	Estimated PopulationProportions% (95% CI) ^1^,^2^
Age			
20–24	66	25	26 (19.0–32.0)
16–19	193	75	74 (68.0–81.0)
School Status			
In School	193	72	74 (72.2–83.1)
Out of School	66	25	26 (16.9–27.8)
Poverty Status			
Abjectly Poor	51	20	21 (16.9–27.2)
Poor	131	51	53 (45.3–58.8)
Non-Poor	72	29	26 (20.1–32.2)
Any IPV past 12 months			
No	33	13	14 (9.3–19.0)
Yes	226	87	86 (81.0–90.7)
Sexual IPV past 12 months			
No	85	33	33 (27.1–38.3)
Yes	172	67	67 (61.7–72.4)
Physical IPV past 12 months			
No	46	18	20 (14.5–25.9)
Yes	211	82	80 (74.1–85.5)
Sexual & Physical IPV past 12 months			
No	102	40	40 (33.6–45.8)
Yes	157	60	60 (54.2–66.4)
Any IPV Frequency past 12 months			
Once	189	73	75 (69.6–80.1)
> Once	70	27	25 (19.9–30.4)
Sexual IPV Frequency past 12 months			
Once	222	86	87 (82.9–90.7)
> Once	37	14	13 (9.3–17.1)
Physical IPV Frequency past 12 months			
Once	199	77	79 (73.4–83.7)
> Once	60	23	21 (16.3–26.6)
Physical & Sexual IPV Frequency past 12 months			
Once	232	90	91 (87.3–93.9)
> Once	27	10	9 (6.1–12.7)

^1^ In the main text only the estimated population proportions are used to describe the results.

^2^ Estimated population proportions were weighted using Respondent Driven Sampling Analysis Tool 5.6

The young women in this survey reported high rates of sexual risk behaviours such as concurrency (87%, CI 77–88), transactional sex (91%, CI 91–96) and age mixing (59%, CI 48–55). These results are reported elsewhere [21]. When we examined associations between IPV and sexual risk behaviours, sexual IPV was significantly associated with age mixing (OR 1.7, CI 1.0–3.1) and transactional sex with the most recent casual partner (OR 2.1, CI 1.1–3.8). Engaging in transactional sex with the most recent casual partner almost doubled the likelihood of having experienced both sexual and physical IPV in the past 12 months (OR 1.8, CI 1.05–3.2). We did not find any statistically significant correlates of physical IPV (Table 2).

**Table 2:** Intimate partner violence and associated sexual risk behaviours among women who have multiple sexual partners.

Explanatory Variables	Sexual IPV OR (95% CI)	Physical IPV OR (95% CI)	Physical & Sexual IPVOR (95% CI)
Age			
20–24 years	1.00	1.00	1.00
16–19 years	0.9 (0.4–1.9)	1.0 (0.4–2.7)	0.5 (0.2–1.6)
Poverty status			
Non-poor	1.00	1.00	1.00
Poor	1.1 (0.5–2.1)	0.7 (0.3–1.9)	1.00
Abjectly poor	1.0 (0.4–2.3)	0.5 (0.1–1.5)	1.2 (0.3–3.6)
School Status			
Out of School	1.00	1.00	1.00
In School	0.5 (0.2–1.2)	0.6 (0.2–1.8)	0.7 (0.2–2.3)
Age mixing in the past 3 months			
No	1.00	***	***
Yes	1.7 (1.00–3.1)		
Transactional sex for money with the most recent casual partner			
No	1.00	***	1.00
Yes	2.1 (1.1–3.8)		1.8 (1.0–3.2)
Sexual Debut			
≥15 years	***	1.00	***
<15 years		1.2 (0.5–2.9)	
Relationship power inequity			
Low	***	1.00	***
High		0.9 (0.4–2.1)	
Number of casual partners in the past 3 months			
≤4 casual partners	***	***	1.00
≥5 casual partners			0.4 (0.2–0.9)
Condom use with casual partners in the past 3 months			
Consistent	***	1.00	1.00
Inconsistent		0.4 (0.2–1.1)	0.4 (0.2–0.9)
Condom use with main partner in the past 3 months			
Consistent	***	***	1.00
Inconsistent			4.0 (0.4–34.1)
Condom use with one night stand at last sex			
No	***	1.00	***
Yes		1.1 (0.5–2.7)	
Male Partner fidelity			
No	***	1.00	1.00
Yes		0.9 (0.4–2.1)	0.6 (0.2–1.7)

*** Predictor variables where p >0.25 in the bivariate analyses were excluded from the final multivariate logistic regression models

To better understand the drivers of the high levels of IPV revealed by the quantitative survey we conducted a qualitative enquiry. The qualitative findings presented below describe the role played by transactional sex, age mixing, young women’s constructions of love, parents and the community in the production of IPV in the study community.

### The role of transactional sex

Consistent with the results from the quantitative analysis that showed a significant association between IPV and transactional sex, in the focus group discussions and individual interviews receiving money or material goods from intimate partners exposed young women to sexual violence:

What happened was, he said to me “listen, I have cigarettes” you see? “let us go and smoke in my shack” you see? And because I am used to going there with him and everyone knows that we normally go to his shack together [I went with him]. So indeed after we’d finished smoking, he wanted to have sex with me. I was like “but no, since when?”…But no [he replied] “listen here, you cannot tell me that, otherwise give me back my smokes (*cigarettes)”* you see? So he beats you up until you give in and do this thing with him (Individual Interview III, 18 years old female)

…he is giving you money and spoils you and things? Well he expects something in return my darling, whether you like it or not, he is going to take it (FGD IV, females aged 16–24)

Facilitator: What if she refuses to go with you at the end?

Participants: (*All talking at once*) Yho! A good *klap* (slap on the face) is coming her way…One klap! (*Laughter*) Just one, she is going to follow you! …. The *skronyonyo* (derogatory term for women who have multiple sexual partners) ate your money, now what? Force, my brother, take it by force!

Participant: As we say “I gave you food, you give me the goods” (*All laughing*) (FGD V, men aged 23–32)

From the quotes above it appears that the exchange of money or gifts placed women at risk of IPV in those relationships where the implicit understanding of the men was that sex would follow the material or monetary exchange.

More worryingly, in some instances young women’s transactional sexual encounters ended in gang rape and murder:

Now when she refused [to go home with the men] they raped her… and threw her over the fence in some yard…they raped her then killed her.” (FGD II, young women aged 16–18)

Similar to the findings of the survey, across all of the focus group discussions, transactional sex severely undermined young women’s sexual power:

Because you are putting a carrot in front of her, she has to listen to you…whatever you want her to do, she will do (FGD 5, men aged 23–32)

He gets to have a hold on you, so that you do not use condoms—he tells you, he does not ask you (FGD III, young women aged 16–24)

Despite the overall portrayal of men in the study community as violent, controlling and exploitative in relations with casual sexual partners, in the men’s focus group discussion, it was pointed out that not all the men in the study community were violent towards their female sexual partners:

…there are the slap giving ones, the take-by-force ones and the don’t-care-move-on-to-the-next-one [type]… (FGD V, men aged 23–32)

### The role of parents and social acceptance of IPV

In addition to sexual risk behaviours, the qualitative findings revealed a particular parenting practice that enhanced young women’s risk of encountering IPV during outings. If young women visited the shebeen at night, most parents were said to not allow them to enter their households if they returned home after their curfew. This meant that young women who transgressed this rule had no option but to go to the home of whomever had bought them alcohol during the shebeen outing, and once at his home, they had no power to control what happened to them:

There is this thing right, you leave your home knowing for a fact that it is strict, and you also know that when you return later, doors will be locked. You tell yourself not to mind, you will find a *slaapplek* (place to sleep) in the end, so you end up going with this person. (FGD III, young women aged 16–24)

Individual interviews revealed that in these conditions, young women’s capacity to escape unwanted sexual attention and sexual violence was limited:

…He slapped me, and I asked “why?” Fine, we wrestled and wrestled; he forced himself on me and had sex with me…What else could I do? I was at his house in Khayelitsha, far away from home and it was around midnight, what could I do? I was even afraid to scream because what if his mother heard me? How would I explain my presence in her house? So ja, you just put up with it… (Individual Interview II, 22 years old female)

Thus, in addition to the masculine power that they accrued from their provision of alcohol and food to the young women during shebeen outings, men engaging in transactional sex also benefited from having their victims in their territory afterwards, at night, a time that is associated with greater likelihood of violence [7].

Worth noting is the fact community members in the study community were said to rarely come to a woman’s aid if she experienced violence at the hands of a man with whom she was known to have a sexual relationship. Not only that, the community judged such a woman harshly if she sought legal recourse:

She cannot lay charges against him, because they are going to say “oh no, but you are with him” they won’t understand what happened, you see? Others will say “but we normally see you going to his place…” so that is how they get away with it…even when you say he has raped you, they will still say “but how is that possible when you are with him?” and when you want them, let’s say to come forward as case witnesses in court, they won’t come forward, they will say “why, it is what you got him used to, why do you not want it all of a sudden?” (Individual Interview VII, 18 years old female)

These beliefs were repeatedly echoed across all five focus group discussions, as young women and men blamed young women for their experiences of violence. There was the belief that women who did not want to be beaten by their partners were outspoken about their intolerance of violence and thus avoided it. The implication from this belief being that those who did not explicitly indicate that they did not want to be physically abused, by their silence, communicated assent:

There are those that don’t like to be beaten and as well maybe the understanding is established in the beginning, and he sees that this girl tells him that I don’t like things like this. (FGD II, women aged 16–18)

### The role of young women’s constructions of love

Some men believed physical violence to be their female partners’ language of love:

You beat them not because you want to, but because that is the only language they understand…they understand love when it is expressed with a beating

(FGD V, men aged 23–32)

Related to this belief, was the idea that young women encouraged physical violence from their steady sexual partners as a way of eliciting affection and apologies for unacknowledged offenses, and to shape younger sexual partners into “real men”:

He is showing you love. He wants to beg you, for you to cry…It’s just the comforting part… its uncommon to experience that kind of comforting way… (FGD II, young women aged 16–18)

He will beat you…you will be crying “hhh, hhh, hhh” and yet you know you could fight back, you know he is your size, but because you want him to be a man, you let him…[because] you also don’t want to be associated with a boy…[so] you toughen him, you make him a man. (FGD IV, young women aged 16–24)

Thus, in this community, IPV was found to be normative and accepted as an inevitable and sometimes necessary social instrument in the negotiation of sex, power and intimacy in different types of sexual relationships.

### Young women’s attitudes towards IPV and male biased sexual norms

Consistent with the revelations of the normalization of IPV in the study community, more than half of the young women (61%, CI 54–68) believed that it was acceptable for a man to hit a woman. More than two-thirds (69%, CI 61–75), believed that men could not control themselves when sexually aroused. However, most of the young women also held other views that did not support sexist gender norms; 85% (CI 80–89) believed that it was acceptable for a woman to refuse sex; two-thirds believed that women should learn to be self-reliant (66%, CI 55–68) and disagreed that men should teach women how to behave (67%, CI 61–72).

### Relationship power inequity in young women’s main partnerships

In the survey, when young women were asked about the distribution of decision-making power and experiences of being controlled in their main partnerships, most of them reported high relationship power inequity; 78% (CI 73–82) and 73% (CI 68–21) reported that their main partners controlled their movements, and monitored where they were at all times. Nearly half of the young women (46%, CI 39–48) reported that if their main partners gave them money or gifts they expected them to do their bidding. More than half (56%, CI 49–62) thought that they would not be able to terminate their relationship with their main partner at will, and nearly two-thirds reported that their main partner had a greater say in decisions affecting the couple (64%, CI 57–68).

However, there were aspects of their sexual relationships where young women shared decision-making power; nearly three quarters (71%, CI 64–76) reported shared decision making power on condom use in their main partnerships, and 70% (CI 64–76) reported that their main partner did not make all of the important decisions in the relationship (Table 3).

**Table 3:** Relationship power equity sample & population proportions among women who have multiple sexual partners.

Item	N	Sample Proportions %	Estimated Population Proportions % (95% CI)
Main partner allows me to greet men I know when we are together			
Yes	136	53	53 (47–59)
No	121	47	47 (40–52)
Main partner wants me at home whenever he comes looking for me			
No	64	25	22 (18–27)
Yes	193	75	78 (73–82)
Main partner is jealous when I look too beautiful			
No	103	40	41 (35–48)
Yes	155	60	59 (51–65)
Main partner has more say in decisions affecting us that I do			
No	160	62	64 (57–68)
Yes	98	38	36 (31–42)
Main partner never controls who I talk to			
Yes	140	54	52 (46–58)
No	118	46	48 (42–54)
I can break up with my main partner anytime I want to			
Yes	124	48	44 (38–50)
No	134	52	56 (49–62)
Main partner does as he wants even if I do not like what he is doing			
No	169	65	65 (60–71)
Yes	89	35	35 (28–40)
Main partner gets his way most of the time when we quarrel			
No	134	52	51 (45–56)
Yes	124	48	49 (43–55)
Main partner always wants to know where I am			
No	70	27	27 (21–32)
Yes	188	73	73 (68–21)
Main partner because he gives me money/gifts he expects me to do everything that he wants me to do			
No	149	58	54 (48–61)
Yes	109	42	46 (39–52)
Main partner makes demands on me			
No	136	58	57 (54–64)
Yes	122	42	43 (40–49)

The qualitative findings confirmed the above; some main partnerships as opposed to casual ones, offered young women the possibility of shared decision making power in some aspects of their relationships;

In some cases it is 50/50, like with your main partner…sometimes you can say to him “like this, like this” (FGD I, women aged 16–21)

Though largely controlled and dominated, it seemed that with main partners young women were more conscious of their voice and their right to have a say in their relationships even if they did not always use them to realise greater relationship power equity:

I think you should tell your boyfriend “I don’t like this, let’s talk about this.

Let’s talk about our rights together”… (FGD III, young women aged 16–24)

…I am very conscious of being taken advantage of, I am very aware of rights and stuff…(Individual interview VII, 18 years old female)

And what I like is to be respected because we are not respected as ladies here… I’m not self-centred but I wish it would be 50/50 when you are in a relationship, if I say this is what needs to happen, it must happen because I need it to be like that, at that time… so when it comes to my rights, Yho!(exclaiming) (Individual interview VI, 16 years old female)

In the men’s focus group discussion, the men also believed that in non-transactional main partnerships, women had some degree of authority and dominance in their relationships:

“The one” is your headmaster (*laughter)*, she is your mother, so you cannot afford to mess up with her, if she says she does not like to be hit, or anything, you will lose her if you do it.

Participant: Yho, especially the pretty ones who make all the men in the location (*township*) go crazy…you know others are just waiting for you to mess up-

Participant: they’ll take her! (FGD V, men aged 23–32)

The above also suggests that due to their commitment and emotional attachment to their main partnerships, some men were less violent towards main female sexual partners than casual sexual partners, for fear of losing them to other men.

Although in the minority, there were men who also believed that the perception that men wielded sole control in their main sexual relationships was nothing but an illusion:

I think it goes both ways, you might think you have control, but she also has control… (FGD V, young men aged 23–32)

Although we did not ask young women about their experiences of power and control in their sexual partnerships with casual sexual partners in the quantitative survey, from the qualitative research presented in this section, it is appears that in casual sexual partnerships marked by transactional sex, women were more controlled and dominated by their male sexual partners.

## Discussion

Our study reports astonishingly high levels of IPV among women who have multiple sexual partners in a peri-urban, Black South African township. Almost 9 out of 10 young women reported being beaten and/or raped, exploited and verbally abused in intimate relationships in the past 12 months. Differences in sampling methodology notwithstanding, when we compare our results to those from studies conducted among young South Africa women in the general population, the levels of IPV found in this study are much higher [5, 8]. For instance, in a study conducted among rural, sexually active young women in the general population in the Eastern Cape Province between 2002 and 2003, 47% and 9% reported experiences of physical and sexual IPV respectively, and 43% reported experiencing both types of IPV in the past 12 months [8]. The findings from our research and those of previous studies that also report multiple sexual partnering as a strong predictor of IPV [5, 8, 10], strongly suggest that in sexual networks characterised by multiple sexual partnerships, many other high risk factors that advance the vulnerability of women to IPV can be expected [14].

The strong associations between IPV, age mixing and transactional sex found in our study send a clear message that among women who have multiple sexual partners, these sexual risk behaviours are inherently structured to undermine women’s power and exacerbate vulnerability to male dominance and abuse [5, 8, 9]. This is because both transactional sex and age mixing introduce dynamics of inequality and dependency in sexual relationships, which provide a social environment that is conducive to the performance of patriarchal models of masculinity that valorise violence [9, 11].

Importantly, several other studies also report transactional sex as a strong predictor of IPV. Among men who have multiple sexual partners in Cape Town, engaging in transactional sex was significantly associated with perpetration of both physical and sexual IPV in the past 12 months [14]. In a study examining associations between sexual risk behaviors and IPV among 1,388 women attending informal drinking establishments in Cape Town, transactional sex was also found to be a strong predictor of IPV, even after controlling for alcohol use [32]. Our qualitative findings confirmed and elucidated the intersection between IPV, age mixing and transactional sex; age and economic asymmetries and the exchange of money and/or gifts boosted male power and sexual entitlement, leading to IPV in instances where young women tried to evade sexual consummation. This context allowed men to treat their younger, poorer female sexual partners as an investment that they owned [14, 8, 11, 33, 34]. We have published the young women’s motivations for engaging in transactional sex elsewhere [31]. The findings on the young women’s motivations indicated that they were driven by both poverty and materialism, in a context that offered them limited access to alternative means of providing for themselves. Thus, economic disempowerment significantly affected and shaped their risk of engaging in transactional sex, how they were treated by their transactional sexual partners and ultimately their risk of experiencing IPV during these sexual encounters.

While violence was experienced across different types of relationships, casual female sexual partners were found to be particularly vulnerable to IPV, owing to the low emotional value that men attached to these relationships and the power and sense of entitlement that the exchange of gifts and money purchased them. The heightened vulnerability of casual sexual partners to IPV in our study is echoed by other studies in the country, which report a strong association between casual, multiple sexual partnering and IPV [13, 14]. The qualitative findings also suggest that casual sexual partnering may have exposed the young women to greater male dominance than main partnerships. These findings echo those of another study conducted among women in the Gauteng province, where casual sexual partnering was associated with all of forms of IPV and relationship power inequity [5].

Although young women reported various forms of relationship power inequity in their main partnerships, there were aspects of their main partnerships where many of them perceived equal decision making power, for instance in the area of condom use and other unspecified areas of decision-making. The men also believed that their female main partners commanded greater respect in their relationships than casual partners. From these findings, it appears that monogamous, non-transactional main partnerships may offer greater opportunities for young women to negotiate shared decision making power with their main partners than transactional, casual relationships.

The high levels of violence that young women experienced in their intimate relationships occurred in a context where physical IPV against women was socially endorsed and accepted with little or no contradiction. Nearly two thirds of the participants in our study believed that it was permissible to hit a woman, a finding very similar to that found in the study conducted among men who have multiple sexual partners in Cape Town [14]. In the qualitative study, the social acceptance and justification of IPV in the study community was repeatedly echoed by statements made in all of the focus group discussions. Some discussions revealed that young women sometimes instigated physical violence to access emotional benefits in their sexual relationships; others suggested that violence was viewed as a love language among young women in the community, and some shared experiences of community bystander apathy, where witnesses of IPV were unresponsive and judgemental towards the victims. These findings are not unique to the study population; across the world, in societies where interpersonal and gender based violence are common, social acceptance and justification of IPV are high [35, 36, 37, 38]. Locally, in a study of young people’s sexual relationships in a South African township in the Eastern Cape, participants named certain forms of IPV, such as sexual coercion, as “township love” and “the way we do things here” [37].

There is much to be understood about the factors that make IPV socially accepted to victims, perpetrators and onlookers; but what has already been established is the critical role played by socio-cultural norms that encourage hierarchical sexual relationships, and valorise aggressive masculinities while promoting timid, subservient performances of femininity [9, 10, 17]. Research shows that the more patriarchal a society, the more difficult it is for men and women to escape its gender biased influence on their relationships, and reject gender norms that oppress and harm women [17]. The power of patriarchal environments to limit women and men’s capacity to fully adopt new, egalitarian and anti-gender based violence relationship norms derives from the fact that in such societies, men and women are held hostage by the roles that their society prescribes for their gender [39], even when they know better. Men and women hold on to these rigid, non-egalitarian gender roles because the social systems that produce them reward adherence and punish defiance against the norm.

The findings of our study also shine a spotlight on violent and domineering male behaviour in the study setting. Scholarship to understand and explain the underlying roots of male aggression in South African society links these to historical and present day socio-political changes. Although these factors were not interrogated in our study, they provide a useful analysis of the historical, cultural and political changes that have shaped the aggressive performances of masculinity reported by young women and men in our study and in other studies conducted in South Africa. Historically, masculine success was defined by land ownership and the ability to establish a homestead in Black African societies in South Africa [40, 41]. However, as the political emasculation of Black men deepened through the colonial and apartheid periods, and as the post-apartheid neo-liberal, economic framework shrunk and casualized the labour market, resulting in the loss of these traditional symbols of masculine success, masculine performances became increasingly compensatory, violent and sexually prodigious [9, 41]. Today, in the absence of legitimate means of displaying masculine success, and to deal with feelings of disempowerment [42], the dominant cultural model of ideal masculinity finds its expression in male performances that dominate women, and celebrate licentious, excessive and aggressive male sexual behaviour [9, 40, 41, 42].

Importantly, as pointed out by participants in this study, men are not a homogenous group; in fact negotiating masculinity in present times in South Africa is complex. In her study of Black, working class men in South Africa, Walker [42] found that “contemporary notions of masculinity in South Africa [are] embryonic, ambivalent and characterized by the struggle between traditional male practices and the desire to be a modern, respectable, and responsible man”.

## Recommendations

The findings of this study highlight the need for strategies that address the intersections between IPV and sexual risk behaviours, rather than only violence or only sexual risk taking. These findings show that where multiple sexual partnering combines with concomitantly high sexual risk behaviours such as age mixing and transactional sex, IPV becomes prevalent and at much higher rates than we find in the general population.

The significant relationship between IPV, transactional sex and age mixing, and the previous findings established by this research [31] indicate the prominent role that was played by gender and economic disempowerment in generating the young women’s vulnerability to both transactional sex and IPV. We thus propose IPV prevention strategies that address not only the prevention of violence in intimate relationships, but also sexual risk reduction and economic and gender empowerment among young women in poor communities. Specifically, anti-gender based violence strategies that strengthen what Kabeer [43] defines as women’s “power to” and “power over” capacities are needed.

Given the findings’ suggestions that the vulnerability of women who are casual rather than main partners to IPV is heightened, strategies should also address and seek to reduce casual sexual partnering by sensitizing populations where this practice is prevalent about its association with gender based violence.

The social acceptance of violence and community bystander apathy in the study community, calls for interventions that emphasise the involvement of community members as allies in efforts to combat gender based violence [44]. Future research needs to determine the viability and effectiveness of community bystander programs, that equip communities to take collective bystander responsibility for the prevention, identification and reporting of incidences of IPV in the community [44].

## References

[1] Abrahams N, Jewkes R, Martin LJ, Mathews S, Vetten L, Lombard C (2009) Mortality of women from intimate partner violence in South Africa: a national epidemiological study. Violence and Victims 24:546–556 19694357 10.1891/0886-6708.24.4.546

[2] Norman R, Schneider M, Bradshaw D, Jewkes R, Abrahams N, Matzopoulos R et al (2009) Interpersonal violence: an important risk factor for disease and injury in South Africa. Population Health Metrics, 8:32 10.1186/1478-7954-8-32 21118578 PMC3009696

[3] Machisa M, Jewkes R, Morne CL, Rama K (2011) The war at home: Findings of the Gender Based Violence Prevalence Study in Gauteng, Western Cape, KwaZulu Natal and Limpopo Provinces of South Africa. Gender Links. Available: http://www.genderlinks.org.za/article/the-war-at-home—-gbv-indicators-project-2011-08-16. Accessed 2012 June 3

[4] Shisana O, Rehle T, Simbayi LC, Parker W, Zuma K, Bhana A et al (2005) South African national HIV prevalence, HIV incidence, behavior and communication survey. Cape Town: Human Sciences Research Council Press Available: http://www.hsrc.ac.za/. Accessed 2007 Jul 27.

[5] Dunkle KL, Jewkes RK, Brown HC, Gray GE, McIntryre JA, Harlow SD (2004) Gender-Based Violence, Relationship Power and Risk of Prevalent HIV Infection among Women Attending Antenatal Clinics in Soweto, South Africa. The Lancet 363: 1415–1421. 10.1016/S0140-6736(04)16098-4 15121402

[6] Pronyk PM, Hargreaves JR, Kim JC, Morison LA, Phetla G, Watts C et al (2006) Effect of a structural intervention for the prevention of intimate-partner violence and HIV in rural South Africa: a cluster randomised trial. The Lancet, 368(9551), 1973–1983. 10.1016/S0140-6736(06)69744-4 17141704

[7] Seedat M, Van Niekerk A, Jewkes R, Suffla S, Ratele K (2009) Violence and injuries in South Africa: prioritising an agenda for prevention. The Lancet 374 (9694): 1011–1022 10.1016/S0140-6736(09)60948-X 19709732

[8] Jewkes R, Dunkle K, Nduna M, Levin J, Jama N (2006) Factors associated with HIV sero-status in young rural South African women: connections between intimate partner violence and HIV International Journal of Epidemiology 2006; 35(60): 1461–8 17008362 10.1093/ije/dyl218

[9] Jewkes R, Morrell R (2010) Gender and sexuality: emerging perspectives from the heterosexual epidemic in South Africa and implications for HIV risk and prevention. Journal of the International AIDS Society 13:6 10.1186/1758-2652-13-6 20181124 PMC2828994

[10] Dunkle K, Jewkes R, Nduna M, Levin J, Jama N, Khuzwayo N et al (2006) Perpetration of partner violence and HIV risk behavior among young men in the rural Eastern Cape. AIDS 20: 2017–14 10.1097/01.aids.0000247582.00826.52 17053357

[11] Luke N, Kurz K (2002) Cross-generational and transactional sexual relations in sub-Saharan Africa. Washington, DC: International Center for Research on Women (ICRW) http://www.icrw.org/files/publications/Cross-generational-and-Transactional-Sexual-Relations-in-Sub-Saharan-Africa-Prevalence-of-Behavior-and-Implications-for-Negotiating-Safer-Sexual-Practices.pdf

[12] Abrahams N, Jewkes R, Laubscher R, Hoffman M (2006) Intimate partner violence: prevalence and risk factors for men in Cape Town, South Africa. Violence and Victims 21(2),247–264 16642742 10.1891/vivi.21.2.247

[13] Petersen I, Bhana A, Mary McKay M (2005) Sexual violence and youth in South Africa: The need for community-based prevention interventions. Child Abuse & Neglect 29:1233–1248 16263168 10.1016/j.chiabu.2005.02.012

[14] Townsend L, Jewkes R, Mathews C, Johnston LG, Flisher AJ, Zembe Y et al (2011) HIV risk behaviours and their relationship to intimate partner violence (IPV) among men who have multiple female sexual partners in Cape Town, South Africa. AIDS and Behavior, 15(1):132–141 10.1007/s10461-010-9680-5 20217470

[15] Jewkes RK, Dunkle K, Nduna M, Shai N (2010) Intimate partner violence, relationship power inequity, and incidence of HIV infection in young women in South Africa: a cohort study. The Lancet, 376(9734), 41–48. 10.1016/S0140-6736(10)60548-X 20557928

[16] Pettifor AE, Measham DM, Rees HV, Padian NS (2004) Sexual power and HIV risk, South Africa. Emerging Infectious Diseases, 10(11) 10.3201/eid1011.040252 PMC3328992 15550214

[17] World Health Organization (2005) WHO multi-country study on women's health and domestic violence against women: summary report of initial results on prevalence, health outcomes and women's responses. Geneva, World Health Organization

[18] South African Police Services (2006) Safety and Security. Available: www.westerncape.gov.za/…/soer_safety_chapter_reuploadedjune06… Accessed 2012 November 28

[19] Shields N, Nadasen K, Pierce L (2008) The effects of community violence on children in Cape Town, South Africa. Child Abuse & Neglect 32(5): 589–601 18511114 10.1016/j.chiabu.2007.07.010

[20] Statistics South Africa (2012) Census in Brief. Pretoria: Statistics South Africa. Report 03-01-41:105. Available: http://www.statssa.gov.za/Census2011/Products/Census_2011_Census_in_brief.pdf. Accessed 2013 June 23

[21] Zembe YZ, Townsend L, Thorson A, Ekstrom AM (2012) Predictors of inconsistent condom use among a hard to reach population of young women with multiple sexual partners in peri-urban South Africa. PLoS One 7 (12) 10.1371/journal.pone.0051998 PMC3527429 23284847

[22] South African Police Services (2012) Crime in Mbekweni (WC) for April to March 2003/2004-2011/2012. Crime Research and Statistics-South African Police Services. Available: www.saps.gov.za/statistics/reports/crimestats/2012/…/mbekweni.pdf. 2012 December 14

[23] Heckathorn D (1997) Respondent driven sampling: a new approach to the study of hidden populations. Soc Probl 44: 174–99.

[24] Chopra M, Townsend L, Johnston L, Mathew C, Tomlinson M, O’Bra H et al (2009) Estimating HIV prevalence and risk behaviors among high-risk heterosexual men with multiple sex partners: use of respondent driven sampling. J Acquir Immune Defic Syndr 51: 72–77. 10.1097/QAI.0b013e31819907de 19282783

[25] Kalichman SC (2010) Social and structural HIV prevention in alcohol-serving establishments: review of international interventions across populations. Alcohol Research & Health 33(3) PMC3860505 23584060

[26] Singh K, Sambisa W, Munyati S, Chandiwana B, Chingono A, Monash R et al (2010) Targeting HIV interventions for adolescent girls and young women in southern Africa: use of the PLACE methodology in Hwange District, Zimbabwe. AIDS and Behavior 14(1): 200–208 10.1007/s10461-009-9572-8 19452272 PMC3966072

[27] Weir SS, Figueroa JP, Byfield L, Hall A, Cummings S, Suchindran C et al (2008) Randomized controlled trial to investigate impact of site‐based safer sex programmes in Kingston, Jamaica: trial design, methods and baseline findings. Tropical Medicine & International Health 13(6):801–813 18482079 10.1111/j.1365-3156.2008.02057.x

[28] Santelli JS, Robin L, Brener ND, & Lowry R (2001) Timing of alcohol and other drug use and sexual risk behaviors among unmarried adolescents and young adults. Family Planning Perspectives 200–205 11589540

[29] Graham K, Bernards S, Abbey A, Dumas T, Wells S (2014) Young women's risk of sexual aggression in bars: The roles of intoxication and peer social status. Drug and Alcohol Review, 10.1111/dar.12153 24844403

[30] Graneheim UH, Lundman B (2004) Qualitative content analysis in nursing research: concepts, procedures and measures to achieve trustworthiness. Nurse Education Today 24(2):105–112 14769454 10.1016/j.nedt.2003.10.001

[31] Zembe YZ, Townsend L, Thorson A, Ekstrom AM (2013) “Money talks, bullshit walks” interrogating notions of consumption and survival sex among young women engaging in transactional sex in post-apartheid South Africa: a qualitative enquiry. Globalization and Health, 9(1):28 23866170 10.1186/1744-8603-9-28 PMC3721991

[32] Pitpitan EV, Kalichman SC, Eaton LA, Sikkema KJ, Watt MH, Skinner D (2012) Gender-based violence and HIV sexual risk behavior: Alcohol use and mental health problems as mediators among women in drinking venues, Cape Town. Social Science & Medicine, 75 (8):1417–1425 22832324 10.1016/j.socscimed.2012.06.020 PMC3425436

[33] Leclerc-Madlala S (2008) Intergenerational/Age-Disparate Sex and Young Women’s Vulnerabilty in Southern Africa. Available: http://www.hsrc.ac.za

[34] Luke N (2005) Confronting the 'Sugar Daddy' Stereotype: Age and Economic Asymmetries and Risky Sexual Behavior in Urban Kenya. International Family Planning Perspectives 31:1 10.1363/3100605 15888404

[35] Kim J, Motsei M (2002) “Women enjoy punishment”: Attitudes and experiences of gender-based violence among PHC nurses in rural South Africa. Social Science & Medicine 54(8):1243–1254 11989960 10.1016/s0277-9536(01)00093-4

[36] Wilson HW, Woods BA, Emerson E, Donenberg GR (2012) Patterns of violence exposure and sexual risk in low-income, urban African American girls. Psychology of Violence, 2(2):194 24563808 10.1037/a0027265 PMC3930142

[37] Wood K, Jewkes R (1997) Violence, rape and sexual coercion: everyday love in a South African township. Gender and Development 5:41–46 12292615 10.1080/741922353

[38] Hindin MJ (2003) Understanding women's attitudes towards wife beating in Zimbabwe. Bulletin of the World Health Organization, 81(7), 501–508. 12973642 PMC2572507

[39] West C, Zimmerman DH (1987) Doing Gender. Gender & Society 1(2): 125–151

[40] Hunter M (2005) Cultural politics and masculinities: Multiple‐partners in historical perspective in KwaZulu‐Natal. Culture, Health & Sexuality 7(4): 389–403 10.1080/13691050412331293458 16864211

[41] Silberschmidt M, Rasch V (2001) Adolescent girls, illegal abortions and “sugar-daddies” in Dar es Salaam: vulnerable victims and active social agents. Social Science & Medicine 52(12):1815–1826 11352408 10.1016/s0277-9536(00)00299-9

[42] Walker L (2005) Men behaving differently: South African men since 1994. Culture, Health & Sexuality 7(3):225–238 10.1080/13691050410001713215 16864199

[43] Kabeer N (2005) Gender equality and women’s empowerment: A critical analysis of the third millennium development goal. Gender & Development 13 (1)

[44] Burn SM (2009). A situational model of sexual assault prevention through bystander intervention. Sex Roles 60 (12):779–792

